# Pericoronary Adipose Tissue Imaging on Coronary CT Angiography: From Fat Attenuation Index to Radiomic Risk Phenotyping

**DOI:** 10.1155/bmri/9341275

**Published:** 2026-09-24

**Authors:** Van Trung Hoang, Vichit Chansomphou, Cong Thao Trinh

**Affiliations:** ^1^ Department of Radiology, Thien Hanh Hospital, Dak Lak, Vietnam, thienhanhhospital.com; ^2^ Department of Radiology, Savannakhet Medical-Diagnostic Center, Kaysone Phomvihane, Laos; ^3^ Department of Radiology, FV Hospital, Ho Chi Minh City, Vietnam

**Keywords:** cardiovascular risk, coronary CT angiography, FAI score, fat attenuation index, pericoronary adipose tissue, pericoronary adipose tissue attenuation, radiomics, reproducibility

## Abstract

Coronary computed tomography angiography (CCTA) has evolved from an anatomical test into a platform for cardiovascular risk phenotyping. Pericoronary adipose tissue (PCAT), which is directly contiguous with the coronary adventitia, changes its composition in response to vascular inflammation and can be interrogated on routine CCTA. Importantly, PCAT attenuation, the fat attenuation index (FAI), and the standardized FAI score are related but nonequivalent measurements: Raw PCAT attenuation is a mean Hounsfield unit measurement within a defined PCAT region, whereas FAI denotes an attenuation‐based methodological construct and FAI score is a standardized, vessel‐specific output adjusted for technical, biological, and anatomical factors. Radiomics extends assessment beyond attenuation by extracting multivariable intensity, shape, and texture features. Selected radiotranscriptomically validated features within the fat radiomic profile (FRP) have been linked to fibrosis and vascularity, but this biological interpretation should not be generalized to all PCAT radiomic signatures. Outcome studies demonstrate clinically relevant associations for attenuation‐based and radiomic biomarkers, although effect sizes, incremental value, and validation differ across populations and analytic platforms. Translation is limited by retrospective evidence, acquisition and reconstruction heterogeneity, lack of universal raw‐attenuation cutoffs, dependence of some standardized outputs on proprietary software, and limited prospective outcome‐driven validation. This narrative review synthesizes the biological rationale, terminology, clinical evidence, technical reproducibility, and emerging technologies in PCAT imaging. We propose a vendor‐neutral reporting and validation framework and position PCAT biomarkers as complementary to coronary calcium, stenosis, plaque phenotype, CT‐derived physiology, and artificial intelligence–assisted risk stratification rather than as a hierarchy of prognostic tests.

## 1. Introduction

Coronary computed tomography angiography (CCTA) now supports evaluation of stenosis, plaque burden and morphology, coronary calcium, and CT‐derived physiological information. Yet anatomical severity alone incompletely captures the biological activity that contributes to plaque destabilization and future coronary events. PCAT has therefore attracted interest as a local imaging sensor of vessel wall inflammation because it lies immediately adjacent to the coronary adventitia and participates in bidirectional paracrine signaling. Translational work demonstrated that inflammatory signals alter adipocyte lipid content and water composition, producing measurable changes in perivascular CT attenuation [1].

**Figure 1:**
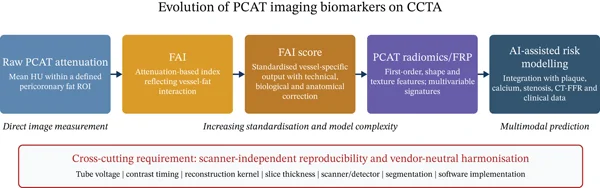
Evolution of PCAT imaging biomarkers on CCTA. Raw PCAT attenuation, FAI, standardized FAI score, radiomic signatures, and AI‐assisted risk models represent related but nonequivalent analytical levels. Radiomic biological interpretation is strongest when directly validated, as in radiotranscriptomic FRP studies. Scanner‐independent reproducibility and vendor‐neutral harmonization are cross‐cutting requirements for translation [1, 3–5].

The literature has expanded rapidly from attenuation‐based measurements to standardized risk scores, radiomics, and artificial intelligence–assisted phenotyping. Recent reviews, including Mátyás et al., emphasize the relationship between PCAT inflammation, plaque vulnerability, and advanced CT characterization [2]. At the same time, heterogeneous terminology has become a source of conceptual and methodological confusion. Raw PCAT attenuation (PCATa), FAI, FAI score, and multifeature radiomic signatures are related but should not be treated as interchangeable biomarkers (Figure 1). Their calculation, technical correction, biological interpretation, and clinical validation differ.

This narrative review therefore has four aims: to clarify terminology, summarize the biological and clinical evidence with explicit attention to the strength and external validation of major studies, identify reproducibility and standardization barriers, and outline how PCAT biomarkers might eventually complement established CCTA reporting and risk stratification tools. The central translational premise is that predictive performance alone is insufficient: A clinically deployable PCAT biomarker must also be reproducible, interpretable, transportable across scanners and populations and demonstrably useful beyond established CCTA markers.

### 1.1. Literature Search and Study Selection

For this narrative review, the PubMed/MEDLINE database was searched for English‐language studies published from January 2017 through August 2026 using combinations of the terms “pericoronary adipose tissue,” “perivascular fat,” “pericoronary adipose tissue attenuation,” “fat attenuation index,” “FAI Score,” “radiomics,” “coronary CT angiography,” “coronary inflammation,” “reproducibility,” and “photon‐counting CT.” In addition, the reference lists of relevant original studies, reviews, and consensus documents were manually screened to identify further publications. Priority was given to biological validation studies, multicenter or longitudinal outcome cohorts, externally validated models, and studies that directly evaluated acquisition or reconstruction effects. Because this is a narrative review, study selection was purposive and no formal PRISMA flow diagram, risk‐of‐bias instrument, or meta‐analysis was performed.

## 2. Biological Basis of PCAT as an Imaging Sensor

**Figure 2:**
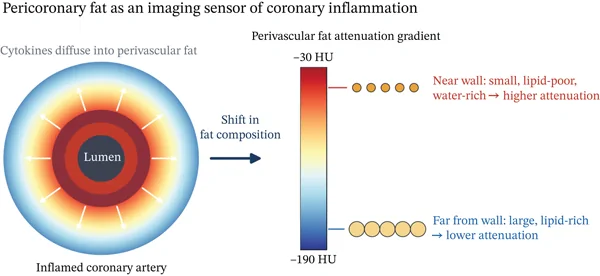
Biological basis of pericoronary adipose tissue imaging. Inflammatory signaling from the coronary wall alters adjacent adipocyte differentiation and lipid‐water composition, producing a spatial shift toward less negative CT attenuation in PCAT. The diagram illustrates the biological basis of attenuation‐based PCAT biomarkers; the numerical measurement may be reported as raw PCAT attenuation or incorporated into a method‐specific FAI implementation [1].

PCAT is the adipose tissue immediately surrounding the coronary arteries without an intervening fascial plane. This anatomical continuity permits signaling between the vessel wall and adjacent adipose tissue. In vascular inflammation, cytokine signaling suppresses local adipogenesis and lipid accumulation. Adipocytes become smaller and relatively water‐rich, shifting CT attenuation toward less negative values within the adipose range [1] (Figure 2). This biology underpins attenuation‐based PCAT imaging, but the measured signal remains an indirect imaging surrogate rather than a histological measurement of inflammation.

The signal is spatially and technically dependent. PCATa differs between coronary territories and varies with vessel size, surrounding anatomy, tube voltage, and other acquisition factors [6, 7]. Consequently, segment selection and measurement conventions matter. The proximal right coronary artery is commonly used because it offers a relatively standardized sampling segment; left coronary measurements may add information but are more vulnerable to short vessel segments, branching anatomy, myocardium, and adjacent structures.

Radiomics can describe intensity distributions and spatial texture beyond a mean attenuation value. The strongest direct biological evidence comes from the 2019 radiotranscriptomic study that correlated selected adipose tissue radiomic features with gene expression markers of inflammation, fibrosis, and vascularity and then developed the FRP [3]. This finding supports a biological interpretation for that specific feature set and model. It does not establish that every PCAT radiomic feature or independently developed radiomic score represents fibrosis or microvascular remodeling. Unless a signature has direct tissue or transcriptomic validation, its biological meaning should be regarded as exploratory.

## 3. Terminology and Attenuation‐Based Measurement

### 3.1. Raw PCATa

PCATa refers to a direct attenuation measurement, typically the mean Hounsfield unit (HU) value of adipose voxels within a predefined pericoronary region. Many studies restrict voxel inclusion to an adipose attenuation window of approximately −190 to −30 HU and define the radial PCAT thickness in relation to vessel diameter. PCATa is therefore a raw image‐derived quantity. It is intuitive and widely accessible, but it is sensitive to tube voltage, reconstruction, contrast conditions, segmentation boundaries, and scanner characteristics [6, 8].

### 3.2. Fat Attenuation Index

FAI was originally developed as an attenuation‐based index of spatial shifts in perivascular fat composition associated with coronary inflammation [1, 9]. In subsequent literature, the term “FAI” has sometimes been used loosely for mean PCATa, while other implementations incorporate spatial or technical correction. To avoid conflation, this review preserves the terminology used by each source. When a study reports an uncorrected mean HU measurement, we use PCATa; when the original study explicitly reports FAI, we retain FAI and describe the measurement context.

### 3.3. FAI Score

FAI score is not synonymous with raw PCATa. It is a standardized vessel‐specific output developed within the CaRi‐Heart platform and adjusted for technical, biological, and anatomical characteristics using reference nomograms [8]. This distinction is clinically important because a threshold derived from raw or study‐specific attenuation cannot be assumed to be equivalent to a standardized FAI score. Proprietary standardization may improve clinical consistency within a validated platform, but it also introduces issues of transparency, access, interoperability, and vendor‐neutral reproducibility.

### 3.4. Radiomic Signatures and FRP

A PCAT radiomic signature is a multivariable model constructed from quantitative image features such as first‐order statistics, shape descriptors, and texture matrices. FRP refers specifically to the machine learning–derived signature developed and biologically interrogated by Oikonomou et al. [3]. FRP should not be used as a generic synonym for any PCAT radiomic model. Throughout this review, “radiomic signature” denotes an independently developed multifeature model unless the study explicitly evaluates FRP.

## 4. From Attenuation to Radiomic Phenotyping

Radiomics converts a segmented PCAT volume into a high‐dimensional quantitative representation. Common feature families include first‐order histogram statistics, shape measures, gray‐level co‐occurrence matrices, gray‐level run‐length matrices, gray‐level size‐zone matrices, and features derived after filtering or resampling [5, 10, 11]. A typical workflow includes coronary centerline extraction, PCAT region definition, attenuation‐based voxel selection, resampling and discretization, feature extraction, stability testing, dimensionality reduction, model training, calibration, and independent validation.

The FRP study illustrates both the promise and the interpretive limits of this approach. In a tissue cohort, selected radiomic patterns showed differential associations with inflammation, fibrosis, and vascularity. In a case‐control development cohort, the resulting FRP achieved an external validation AUC of 0.77; in 1575 SCOT‐HEART participants followed for a median of 4.8 years, FRP provided prognostic information beyond clinical risk factors, calcium score, stenosis, and high‐risk plaque features [3]. The biological inference is therefore strongest for this specific radiotranscriptomically anchored model.

Other PCAT radiomics studies have reported discrimination of acute myocardial infarction, prediction of acute coronary syndromes, and prediction of rapid plaque progression [12–14]. These models are potentially useful but remain heterogeneous in segmentation, preprocessing, feature definitions, outcome selection, and validation. Their constituent features should not automatically be interpreted as histological fibrosis, angiogenesis, or inflammation without direct biological validation.

High‐dimensional modeling also creates statistical vulnerability. Feature selection performed before cross‐validation, data leakage, small event counts, scanner‐specific texture, and inadequate calibration can generate optimistic performance estimates. Feature stability should therefore be assessed before model development, and external validation should test not only discrimination but also calibration, clinical net benefit, and transportability [5, 15].

## 5. Clinical Evidence and Applications

### 5.1. Cross‐Sectional Disease Activity and Culprit‐Lesion Assessment

Cross‐sectional studies show that PCATa varies across stages of coronary disease and can be higher around high‐risk or culprit lesions [7, 16]. These observations support the biological concept of local vessel‐fat crosstalk, but they do not by themselves establish prospective risk prediction. In acute coronary syndromes, measurements may also be influenced by acute edema, treatment, motion, and contrast conditions. Culprit‐lesion applications therefore remain investigational.

### 5.2. Plaque Progression

Serial CCTA provides a mechanistic bridge between baseline PCAT phenotype and subsequent anatomical change. In a 2024 two‐center retrospective study of 1233 patients undergoing serial CCTA, a seven‐feature PCAT radiomics model predicted rapid plaque progression with AUCs of 0.85, 0.84, and 0.81 in the training, internal validation, and external validation cohorts, respectively [14]. Importantly, adding clinical or plaque‐characteristic models to the radiomics model did not significantly further improve discrimination. This result supports external transportability within that study design but also illustrates why incremental value over established CCTA markers should be tested rather than assumed.

### 5.3. Outcome Prediction and Incremental Value

The CRISP‐CT study provided early large‐scale prognostic evidence. Across 3912 patients in derivation and validation cohorts, higher perivascular FAI was independently associated with cardiac mortality. A study‐specific threshold near −70.1 HU identified higher risk groups, but this value should not be interpreted as a universal raw‐attenuation cutoff because scanner, protocol, reconstruction, and postprocessing differences can shift attenuation measurements [6, 8, 9].

The ORFAN multicenter longitudinal study substantially expanded the outcome evidence in patients without obstructive coronary disease. In a registry of 40,091 consecutive patients from eight hospitals, most patients had nonobstructive disease, yet this group accounted for the majority of major adverse cardiac events and cardiac deaths; in a deeply phenotyped cohort of 3393 patients, vessel‐specific FAI score was strongly associated with long‐term cardiac outcomes [17]. Because ORFAN evaluated a standardized FAI score rather than a simple raw mean attenuation, its effect estimates should not be extrapolated directly to PCATa measured with unrelated software.

Recent prospective evidence is encouraging but indicates that incremental gains may be modest when modern CCTA markers are already available. In 1029 patients with Type 2 diabetes followed for a median of 56.5 months, RCA PCATa independently predicted major adverse cardiovascular and cerebrovascular events (adjusted hazard ratio 1.86, 95% CI 1.24–2.80). Adding PCATa to a model containing clinical variables, conventional CCTA findings, high‐risk plaque, and CT‐derived fractional flow reserve increased AUC from 0.73 to 0.75 [18].

Conversely, the 2025 ICONIC matched case‐control analysis provides an important counterpoint. Among 400 patients, mean PCATa did not differ significantly between those with future acute coronary syndromes and controls (−72.99 ± 9.42 HU vs. −73.96 ± 9.47 HU; *p* = 0.08), although PCATa remained weakly associated with incident events after adjustment for plaque volume, stenosis, and high‐risk plaque features (adjusted hazard ratios approximately 1.014–1.015 per HU) [19]. These findings argue against portraying PCAT biomarkers as uniformly superior to established CCTA markers.

Taken together, the evidence supports a complementary rather than hierarchical framework. Calcium burden, luminal stenosis, plaque phenotype, ischemia, PCAT inflammation, and radiomics measure different dimensions of coronary disease. Incremental clinical value should therefore be demonstrated in the population and workflow in which the biomarker will be used. Table 1 directly compares representative studies, including population, follow‐up, endpoint, metric, quantitative result, and validation status.

**Table 1:** Selected studies illustrating biological validation, clinical outcomes, radiomic prediction, and technical reproducibility of PCAT imaging.

Study/design	Population and follow‐up	Metric	Endpoint/key quantitative result	Validation and interpretation
Antonopoulos et al., 2017 [1]	Translational cardiac surgery tissue and CCTA cohorts	Perivascular attenuation/FAI concept	Linked vessel inflammation to local adipocyte composition and CT attenuation changes.	Direct biological validation established the vessel‐fat attenuation mechanism.
Oikonomou et al. (CRISP‐CT), 2018 [9]	3912 patients; median follow‐up approximately 72 months (derivation) and 54 months (validation)	FAI (study terminology)	Higher RCA FAI independently predicted cardiac mortality; study‐specific threshold near −70.1 HU identified high‐risk groups.	Independent derivation and validation cohorts; threshold should not be treated as scanner‐independent.
Oikonomou et al. (FRP), 2019 [3]	167 tissue biopsies; 202 case‐control subjects for model development; 1575 SCOT‐HEART participants, median 4.8 years	FRP radiomic signature	External validation AUC 0.77; FRP remained prognostic beyond clinical risk, calcium, stenosis, and HRP in SCOT‐HEART.	Radiotranscriptomic biological anchoring plus external/model‐wide validation; fibrosis/vascularity inference applies specifically to validated features.
Chan et al. (ORFAN), 2024 [17]	40,091 consecutive patients in Cohort A; deeply phenotyped Cohort B *n* = 3393; long‐term follow‐up	FAI score	Standardized vessel‐specific inflammatory risk strongly associated with cardiac mortality and MACE, including nonobstructive CAD.	Large multicenter longitudinal evidence; metric is standardized FAI score, not raw mean PCATa.
Li et al., 2024 [14]	1233 patients from two centers with serial CCTA > 6 months; training, internal, and external validation cohorts	PCAT radiomic signature	RPP prediction AUC 0.85 training, 0.84 internal validation, 0.81 external validation.	External validation present; combining clinical/plaque models with radiomics did not significantly further improve AUC.
Ding et al., 2025 [18]	Prospective single‐center cohort, *n* = 1029 with Type 2 diabetes; median 56.5 months	RCA PCATa	MACCE: adjusted HR 1.86 (95% CI 1.24–2.80); AUC increased from 0.73 to 0.75 after adding PCATa to clinical + conventional/advanced CCTA model.	Prospective evidence; incremental discrimination was statistically significant but modest.
Kwan et al. (ICONIC), 2025 [19]	Matched multicenter case‐control analysis, 200 future ACS cases + 200 controls; 1174 vessels	PCATa	Mean PCATa: −72.99 ± 9.42 vs. −73.96 ± 9.47 HU (*p* = 0.08); adjusted HR ~1.014–1.015 per HU in survival models.	Provides a balanced counterpoint: independent association but limited group separation and modest single‐marker utility.
Lisi et al., 2025 [8]	Technical CCTA reconstruction study	PCAT attenuation	PCAT attenuation changed substantially with reconstruction kernel and iterative‐reconstruction settings.	Supports protocol standardization before applying thresholds or pooling multicenter data.

Abbreviations: ACS, acute coronary syndrome; AUC, area under the curve; CAD, coronary artery disease; CCTA, coronary CT angiography; FAI, fat attenuation index; FRP, fat radiomic profile; HR, hazard ratio; HRP, high‐risk plaque; MACCE, major adverse cardiovascular and cerebrovascular event; MACE, major adverse cardiac event; PCAT, pericoronary adipose tissue; PCATa, pericoronary adipose tissue attenuation; RCA, right coronary artery; RPP, rapid plaque progression.

## 6. Reproducibility Barriers to Translation

PCAT biomarkers are derived from CT images, and CT images are products of both biology and imaging physics. Tube voltage affects tissue attenuation and the distribution of voxels within a fixed adipose window; contrast timing and partial volume effects alter perivascular measurements; and reconstruction kernel, slice thickness, iterative reconstruction strength, and deep‐learning reconstruction modify image noise and texture [6, 8]. These effects are particularly relevant when published thresholds or radiomic models are transferred between institutions.

Reconstruction is a major concern for radiomics because texture features may be more sensitive to noise and spatial resolution than mean attenuation. The 2025 technical study by Lisi et al. showed that PCATa itself changes substantially with reconstruction kernel and iterative reconstruction settings, reinforcing the need for protocol control even before high‐dimensional texture analysis is attempted [8].

Segmentation adds observer‐ and algorithm‐dependent variability. Small differences in coronary centerline tracking or radial boundaries can include myocardium, cardiac chambers, veins, calcification, stents, or nontarget epicardial fat. Manual segmentation is labor‐intensive and introduces interobserver variation, while automated approaches may fail in motion‐degraded, heavily calcified, stented, or anatomically complex vessels. Repeatability and interobserver assessment should therefore be reported for both attenuation metrics and candidate radiomic features [20].

Software implementation is another source of hidden heterogeneity. Resampling, interpolation, gray‐level discretization, filter definitions, and feature equations may differ across packages. IBSI‐compliant feature definitions and explicit software versioning improve reproducibility, but standardization of feature extraction does not eliminate variability introduced upstream by acquisition and segmentation [5, 21].

Accordingly, scanner‐independent and vendor‐neutral harmonization should be considered a prerequisite for broad multicenter deployment. A useful minimum validation package includes repeated reconstruction or test–retest analysis, interobserver and intraobserver segmentation assessment, prespecified stability thresholds, sensitivity analysis across major acquisition settings, and independent external validation. Predictive but unstable features should not enter a clinical model.

## 7. Harmonization and Reporting Standards

Harmonization can be addressed at acquisition, image, and feature levels. Acquisition harmonization is conceptually preferable but often impractical in retrospective multicenter datasets. Image‐domain approaches attempt to reduce scanner‐related appearance differences before feature extraction. Feature‐domain approaches such as ComBat adjust feature distributions for batch effects and can improve cross‐center comparability [22, 23]. However, statistical harmonization is not a substitute for biological and technical validation; inappropriate covariate specification can remove clinically meaningful signal or preserve confounding.

Transparent reporting is therefore essential. Studies should document scanner vendor and model, acquisition mode, tube voltage, tube current strategy, ECG gating, contrast protocol, reconstruction kernel, slice thickness, reconstruction algorithm, cardiac phase, coronary segment and length, radial sampling rule, attenuation window, segmentation method, preprocessing, radiomics software, feature‐stability criteria, feature‐selection strategy, validation design, calibration, and decision‐curve analysis where relevant. The proposed checklist in Table 2 distinguishes items grounded in existing standards from PCAT‐specific recommendations proposed by the authors. IBSI informs radiomics feature definition and preprocessing, while TRIPOD principles inform transparent development and validation of multivariable prediction models [5, 15].

**Table 2:** Reproducibility‐oriented reporting checklist for PCAT attenuation and radiomics studies, with explicit distinction between existing standards and author‐proposed PCAT‐specific recommendations.

Domain	Minimum items to report	Why it matters	Basis
CCTA acquisition	Scanner vendor/model; detector configuration; acquisition mode; tube voltage; tube current strategy; ECG gating; contrast volume/timing; cardiac phase.	Defines the imaging system and contrast conditions that influence attenuation and texture.	PCAT technical literature + authors′ PCAT‐specific minimum recommendation.
Reconstruction	Kernel; slice thickness/increment; iterative or deep‐learning reconstruction type and strength; field of view.	Reconstruction modifies HU, noise, spatial resolution, and radiomic texture.	PCAT reconstruction evidence [8] + authors′ recommendation.
PCAT definition	Coronary artery/segment; analyzed length; radial sampling rule; attenuation window; exclusion rules.	Allows replication of the anatomical ROI and voxel selection.	Published PCAT conventions [1, 9] + authors′ recommendation.
Segmentation	Manual, semiautomated, or automated method; software/version; observer training; quality control; failure cases.	Boundary and centerline differences affect attenuation and texture.	Repeatability evidence [20] + authors′ recommendation.
Preprocessing	Resampling; interpolation; filtering; discretization; bin width/bin number; intensity normalization if used.	Radiomic features can change materially with preprocessing choices.	IBSI‐based reporting [5].
Feature extraction	Software/toolbox and version; feature classes; IBSI compliance; custom feature definitions.	Prevents ambiguity in mathematical feature definitions.	IBSI [5] and software transparency [21].
Feature stability	Interobserver/intraobserver analysis; test–retest or repeated reconstruction; prespecified ICC/stability threshold; sensitivity across key settings.	Unstable features should be excluded before model training.	Authors′ PCAT‐specific minimum recommendation informed by reproducibility literature.
Model development	Outcome definition; feature selection; algorithm; nested cross‐validation; class imbalance; missing data; hyperparameter tuning.	Reduces leakage and overfitting and improves reproducibility.	TRIPOD principles [15] + authors′ radiomics‐specific extension.
Validation	Independent external cohort; scanner/protocol diversity; discrimination; calibration; confidence intervals; decision‐curve analysis when appropriate.	Tests transportability and clinical utility, not just internal performance.	TRIPOD [15] + authors′ PCAT‐specific recommendation.
Harmonization	Level of harmonization; batch variable; protected biological covariates; training‐only fitting of harmonization parameters; sensitivity analysis.	Harmonization can reduce scanner effects but can also remove true signal.	ComBat guidance [22, 23].
Clinical integration	Reference model and incremental value over calcium, stenosis, plaque burden/HRP, and CT‐derived FFR when available; management consequence.	Clarifies whether PCAT adds actionable information to contemporary CCTA.	Authors′ proposed clinical‐translation minimum; CAD‐RADS context [24].

Abbreviations: CCTA, coronary CT angiography; FFR, fractional flow reserve; HRP, high‐risk plaque; HUs, Hounsfield units; IBSI, Image Biomarker Standardisation Initiative; ICC, intraclass correlation coefficient; PCAT, pericoronary adipose tissue; ROI, region of interest; TRIPOD, Transparent Reporting of a multivariable prediction model for Individual Prognosis Or Diagnosis.

The purpose of the checklist is not to prescribe a single universal PCAT protocol. Rather, it makes technical heterogeneity visible and auditable. This is particularly important when readers judge whether a published threshold or model can be transported to a local scanner, reconstruction pipeline, population, or software environment.

## 8. Limitations of the Current Evidence

Several limitations constrain the interpretation and clinical generalizability of current PCAT literature. First, much of the evidence is retrospective, post hoc, or case‐control in design. Prospective outcome‐driven studies exist, including recent work in diabetes, but prospective multicenter validation remains limited relative to the breadth of proposed applications [18]. Second, acquisition and reconstruction protocols vary substantially across studies, and PCATa and radiomic texture are sensitive to scanner and reconstruction settings [6, 8].

Third, no universally accepted scanner‐independent raw PCATa cutoff exists. Study‐specific thresholds can be prognostically useful, but they should not be transferred across protocols without validation. Standardized FAI score attempts to address part of this problem through technical and biological adjustment, but it is implemented within proprietary software and therefore does not by itself solve vendor‐neutral reproducibility or access [4]. Fourth, outcome definitions, coronary segments, attenuation metrics, radiomics pipelines, and adjustment models differ, limiting direct comparison and meta‐analytic synthesis.

Fifth, many radiomic studies remain vulnerable to overfitting, dataset shift, and limited external validation. Biological interpretation is often inferred from feature labels or associations rather than directly validated against tissue, and such interpretations should be considered exploratory outside radiotranscriptomically validated models [3]. Finally, prognostic association does not establish actionability. Evidence is still insufficient to conclude that changing treatment specifically because of a PCAT biomarker improves patient‐centered outcomes. Prospective implementation or interventional studies are needed before routine therapy escalation is justified on PCAT findings alone.

## 9. Future Directions and Clinical Implementation

### 9.1. Integration With Contemporary CCTA Reporting

Future clinical use should position PCAT biomarkers alongside, not above, established CCTA domains. CAD‐RADS 2.0 provides a standardized framework for reporting stenosis, plaque burden, high‐risk plaque, and ischemia‐related modifiers [24]. PCAT is not currently a formal CAD‐RADS modifier. A reasonable research pathway would therefore evaluate a separately reported inflammatory phenotype that complements the CAD‐RADS category, coronary calcium, quantitative plaque burden and composition, and CT‐derived fractional flow reserve. Such integration should be tested for calibration, reclassification, decision‐curve benefit, and impact on management rather than judged only by AUC.

**Figure 3:**
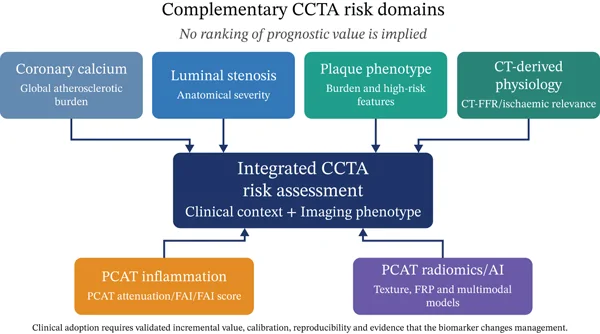
Complementary CCTA risk domains. Coronary calcium, stenosis, plaque phenotype, CT‐derived physiology, PCAT inflammation, and PCAT radiomics/AI are presented as complementary information domains rather than a hierarchy of progressively increasing prognostic value. PCAT is not currently a formal CAD‐RADS 2.0 modifier; future integration should be evaluated as an adjunct to established reporting and risk stratification components [24].

This complementary approach is also consistent with contemporary chronic coronary syndrome management, in which anatomical burden, physiological significance, symptoms, and global clinical risk are integrated rather than reduced to a single imaging marker [25]. Figure 3 therefore presents PCAT inflammation and radiomics as parallel information domains within an integrated CCTA risk model, without implying a monotonic ranking of prognostic value.

### 9.2. Automation, Interpretability, and Vendor‐Neutral Deployment

Automated coronary centerline extraction and PCAT segmentation are likely required for practical clinical scaling. However, automation must include quality control and explicit failure handling for motion, calcification, stents, bypass grafts, variable contrast, and anatomical variants. For radiomic or AI models, interpretability should include calibration, feature stability, uncertainty, and clinically meaningful risk categories. External validation should span scanner vendors, reconstruction methods, geographic regions, and demographic groups before a model is considered transportable.

### 9.3. Photon‐Counting CT

Photon‐counting detector CT (PCD‐CT) is a particularly relevant emerging technology because it changes spatial resolution, noise properties, and spectral information compared with conventional energy‐integrating detector CT. Early phantom and intraindividual studies demonstrate that adipose attenuation, FAI‐type measurements, and PCAT radiomic features can differ between detector technologies [26–28]. PCD‐CT may ultimately improve small vessel and perivascular characterization, but legacy PCAT thresholds and radiomic models should not be assumed to transfer unchanged. Technology‐specific calibration, reconstruction harmonization, and multicenter reproducibility testing will be necessary.

### 9.4. Prospective Clinical Utility

The most important next step is prospective clinical utility testing. Multicenter studies should determine whether adding PCAT biomarkers to optimized CCTA interpretation meaningfully changes preventive therapy, downstream testing, or follow‐up and whether these changes improve outcomes. Comparative studies should predefine the reference model, including calcium, stenosis, plaque burden and high‐risk features, and CT‐derived physiology where available. Only then can the field determine whether PCAT imaging functions as a useful independent biomarker, a complementary risk modifier, or a research phenotype in specific clinical settings.

## 10. Conclusion

PCAT imaging provides a biologically plausible window into the local coronary microenvironment, but its biomarkers require precise terminology and evidence‐proportionate interpretation. Raw PCATa, FAI, standardized FAI score, and radiomic signatures are related but nonequivalent. Radiomics can capture information beyond mean attenuation, yet fibrosis and microvascular‐remodeling interpretations are directly supported only for selected biologically validated signatures and should not be generalized to all models.

Clinical studies support prognostic and disease‐characterization value, including large multicenter cohorts and recent prospective evidence, but incremental performance varies by population and analytic method. Translation is currently limited by protocol heterogeneity, scanner and reconstruction dependence, lack of universal raw‐attenuation cutoffs, proprietary implementations, radiomic instability, and insufficient prospective outcome‐driven validation. The most credible path to adoption is therefore complementary integration with established CCTA markers, vendor‐neutral standardization, transparent reporting, rigorous external validation, and demonstration that PCAT‐guided information changes clinically meaningful decisions.

## Author Contributions

Van Trung Hoang: conceptualization, methodology, visualization, writing—original draft, and writing—review and editing. Vichit Chansomphou: investigation, validation, project administration, and writing—review and editing. Cong Thao Trinh: investigation, validation, and writing—review and editing.

## Funding

No funding was received for this manuscript.

## Disclosure

All authors reviewed and approved the final version of the manuscript.

## Ethics Statement

The authors have nothing to report. This is a narrative review based on published literature and does not involve human participants, patient data, or animal experiments.

## Consent

The authors have nothing to report.

## Conflicts of Interest

The authors declare no conflicts of interest.

## Data Availability

Data sharing is not applicable because no datasets were generated or analyzed in this review.
